# Location, Location, Location: Geographic source of blacklegged tick (*Ixodes scapularis*) nymphs determines behavioral outcomes in laboratory studies

**DOI:** 10.21203/rs.3.rs-7312978/v1

**Published:** 2025-09-10

**Authors:** Karen C. Poh, Jessica E. Brown, Mia I. Esoldo, Erika T. Machtinger

**Affiliations:** Pennsylvania State University; Pennsylvania State University; Pennsylvania State University; Pennsylvania State University

**Keywords:** Behavior, parasite, Lyme disease, infestation, genotypes, ticks

## Abstract

*Ixodes scapularis* is considered a significant medical and veterinary arthropod pest, capable of transmitting several pathogens that cause disease in humans and animals. Previous work has identified two distinct populations of *I. scapularis* in the United States (northern and southern), characterized by differences in their genetics and behavior. This study aimed to characterize and compare the lateral movement and feeding behaviors of nymphal *I. scapularis* between the northern and southern populations in the United States. Using laboratory-reared ticks from BEI (northern), Oklahoma State University (southern), and field-collected ticks from central Pennsylvania (Mid-Atlantic), behavioral bioassays were conducted to quantify distances traveled and velocities in a one-hour time frame. Ticks from the northern lineage walked longer distances and at faster speeds compared to ticks from the southern lineage. Field-collected ticks from central Pennsylvania, located between what is considered the northern and southern populations, exhibited similar movement behaviors as ticks from the southern population, even though ticks from the Mid-Atlantic are geographically categorized as the northern population. To compare feeding behaviors, colony-reared white-footed mice (*Peromyscus leucopus*) were artificially infested with northern and southern ticks, and the percentages of infestation and feeding successes were compared. Northern ticks had higher success in infestations and feeding to repletion compared to southern ticks. These behavioral differences in movement and feeding patterns provide additional evidence for the hypothesis that geographically distinct populations of *I. scapularis* exist across the United States. Researchers should consider these population differences when selecting tick lineages for behavioral studies and other blacklegged tick research.

## Introduction

The blacklegged tick (*Ixodes scapularis* Say, Acari: Ixodidae) is considered the most significant vector of tick-borne pathogens in North America, capable of transmitting *Borrelia burgdorferi* (Lyme disease), *Anaplasma phagocytophilum* (human granulocytic anaplasmosis), *Babesia microti* (human babesiosis), and Powassan virus (Eisen and Eisen 2018). Although blacklegged ticks are found throughout the northeastern, Mid-Atlantic, northcentral, and southern United States, these regions have varying levels of reported tick-borne diseases associated with the blacklegged tick (Rosenberg et al. 2018). Differences in disease incidence may be related to variations in the ecology of *I. scapularis*, resulting from differing demographic histories in their respective geographic regions (Norris et al. 1996, Ginsberg et al. 2014, Sakamoto et al. 2014, Arsnoe et al. 2015, 2019).

The blacklegged tick comprises at least two distinct populations: northern and southern (Norris et al. 1996, Van Zee et al. 2013, Sakamoto et al. 2014). These populations exhibit notable differences in genetics, survival patterns, host attachment preferences, and questing behavior. Numerous studies have documented these regional variations in *I. scapularis* biology and ecology (Piesman and Spielman 1979, Carey et al. 1980, Mather et al. 1989, Apperson et al. 1993, Goddard 1993, Norris et al. 1996, Durden et al. 2002, Piesman 2002, LoGiudice et al. 2003, Goddard and Piesman 2006, Van Zee et al. 2013, Ginsberg et al. 2014, Sakamoto et al. 2014, Arsnoe et al. 2015, 2019, Curtis et al. 2020, Tietjen et al. 2020).

Understanding the differences between the two distinct lineages of blacklegged ticks could have important implications for prediction, prevention, and control. For example, information on questing and horizontal movement could potentially improve tick bite risk models by accounting for differences in movement per lineage (Hassett et al. 2022, Marshall et al. 2025). Furthermore, understanding the feeding patterns of different tick populations on reservoir hosts could clarify predictions about tick-borne disease risk.

Questing behavior (vertical movement) differs between *I. scapularis* populations in the United States, with northern populations questing more frequently and at greater heights than their southern counterparts (Arsnoe et al. 2015, 2019, Tietjen et al. 2020). While lateral movement has been evaluated separately for adult *I. scapularis* from Mississippi and New York (Goddard 1993, Curtis et al. 2020), direct comparisons of horizontal movement between northern and southern lineages remain lacking, particularly for nymphal stages. Similarly, potential behavioral differences in host infestation and feeding success between these populations have not been systematically assessed.

When evaluating tick behavior, it is important to consider the impact of the source population. Therefore, our study aims to identify differences in movement and feeding behavior of nymphal blacklegged ticks from these two distinct lineages. This study compared the horizontal movement (distance) and speed (velocity) of nymphal *I. scapularis* from northern and southern lineages, as well as an additional lineage that originates from an area that lies between the northern and southern populations (central Pennsylvania). Host infestation and feeding differences between northern and southern lineages were also evaluated.

## Materials and Methods

### Movement Assessment

For this study, *I. scapularis* that are found in the northeast and central regions of the United States north of the Chesapeake Bay are considered the “northern” population, and ticks found south of this region are the “southern” population (Ginsberg et al. 2014). Nymphal *I. scapularis* ticks were acquired from three locations: (1) BEI Resources (ATCC, Manassas, VA), which was started with ticks collected in Rhode Island (northern ticks); (2) Oklahoma State University Tick Lab (Stillwater, OK), which was started using ticks collected in Oklahoma (southern ticks); and (3) several field locations in central Pennsylvania (Centre County, PA) (Mid-Atlantic ticks). After ticks were received or collected, they were stored in snap cap vials with modified lids and mesh to allow airflow (Levin and Schumacher 2016). Vials of ticks were then stored in a humidity chamber at 80–95% RH. The chamber was housed in an environmental chamber set at 22°C and a L:D setting of 16:8 (Troughton and Levin 2007, Levin and Schumacher 2016).

Once ready for observation in the experiment, individual ticks were removed from their vials using a pair of featherweight forceps or a paintbrush. To ensure ticks were alive before the start of the experiment, ticks that were actively moving in the vial were chosen. During each bioassay, ticks were observed between 0800 and 1300 h (Schulze et al. 2001, Schulze and Jordan 2003). A similar temperature (20.3 ± 0.8°C) and relative humidity (85.5 ± 6.1%) were maintained for the bioassay using a portable heater and humidifier, respectively, within the humidity chamber.

One nymphal tick lineage was randomly chosen for observation for each replicate. A tick was placed into a 100 × 15 mm glass petri dish (Pyrex, Corning, Inc., Glendale, AZ) and was given approximately five minutes to acclimate before recording began. Tick movement was recorded using the Basler acA1300–60gm GigE camera (Basler AG, Ahrensburg, Germany) at a frame rate of 30 frames per second and a resolution of 1280 × 1024 pixels. The camera feed was connected to a laptop running MediaRecorder 6 (Noldus, Wageningen, the Netherlands), which recorded and stored the video. The video recording was then imported into EthoVision XT 15 (Noldus, Wageningen, Netherlands) to quantify movement. After acclimation, the camera recording was started, and tick movement was tracked for one hour. Once completed, the tick was removed and not reused. While ticks that seemed active in the vials were chosen for the experiment, the tick was stimulated at the end of the experiment by breathing air to check that the ticks did not expire while being recorded. If ticks did not respond, the recording was not used. The petri dish was washed with unscented soap and water, dried, and then replaced under the camera. Multiple replicates were completed in one day, with all experiments ending by 1300 h. Preliminary trials of the experiment revealed that ticks reduced or completely stopped their movement after approximately 2.5–3 hours of the experiment starting, which would skew average velocity calculations.

Before being imported into EthoVision, recordings were reviewed to ensure no artifact movements (i.e., movements other than the tick) were detected and that the camera maintained a clear view of the tick for the entire observation period. If either occurred, the recording was not used in the analysis. Once the videos passed inspection, they were imported into EthoVision XT 15 and analyzed by the software, which calculated the total distance the tick moved (in cm) and its average velocity (in cm/s). After removing videos with artifacts or obscured views, 60 replicates of each of the northern and Mid-Atlantic lineages and 58 replicates of the southern lineage were included in analyses.

Total distances and average velocities of each tick population were compared using a one-way ANOVA, calculated using the *aov* package in the statistical program R (R Core Team 2024). Post-hoc pairwise comparisons were conducted using Tukey’s HSD. Significance was set at α = 0.05.

### Tick Feeding Behavior Assessment

Mice were artificially infested with ticks to examine differences in tick infestation and feeding patterns between the northern and southern lineages of *I. scapularis*. White-footed mice (*Peromyscus leucopus*) between the ages of 3–24 months were used in the experiment. Animals used were from the F1 and F2 generations of an in-house, wild-caught colony of *Peromyscus leucopus* and housed according to approved mouse-rearing standards of The Pennsylvania State University. Housing and experimental procedures were conducted according to approved Institutional Animal Care and Use Committee (IACUC) protocols (PROTO202101808, PROTO202001598) and met the requirements of the Public Health Service Policy on Humane Care and Use of Laboratory Animals. A total of 66 animals (33 males, 33 females) were used in the experiments and included in the statistical analyses. In infestations involving northern ticks, 21 male and 23 female mice were used, while infestations with southern ticks used 12 male and 10 female mice. Because pathogens within field-collected ticks could not be verified before infestation, field-collected tick testing was not approved by the IACUC; thus, only northern and southern lineages of nymphal *I. scapularis* were used for the tick feeding behavior assessment.

In each round of infestations, three mice were infested with ticks of either lineage as part of complementary projects evaluating rodent behavior to tick infestation. Infestation procedures followed Brown et al. (2025). To infest mice, nymphal ticks were added to white cotton crew socks, which were held open by small plastic cups. The number of ticks used varied based on lineage-specific attachment rates and experimental requirements. For the northern lineage, 15–30 ticks were used per sock, while for the southern lineage, 30–60 ticks were used per sock due to consistently lower attachment rates observed in preliminary trials. All socks in one round were infested with the same lineage of ticks.

After mice were anesthetized and added to the sock, a square metal bulldog clip was used to close the sock. The cups holding the socks were then moved to secondary containment. The ticks were given three hours to infest the mice before the mice and attached ticks were moved to Noldus Phenotyper cages (Model PT3000; Noldus, Wageningen, Netherlands). Ticks remaining in the sock were counted and subtracted from the original number added (depending on the lineage) to obtain the approximate number of ticks that successfully infested the mice. The percentage of successful infestation was calculated as the number of ticks that infested mice divided by the original number of ticks added to the sock multiplied by 100.

Mice were observed in the Phenotyper cages for four days, and the number of replete nymphs was counted daily. The percentage of successful feeding was calculated as the total number of replete ticks divided by the number of ticks that successfully infested mice, expressed as a percentage, and then multiplied by 100. Wilcoxon signed rank tests were performed in R to determine if the percentages of successful infestation and successful feeding differed between the northern and southern lineages of *I. scapularis*. Significance was set at α = 0.05.

## Results

**Figure 1:**
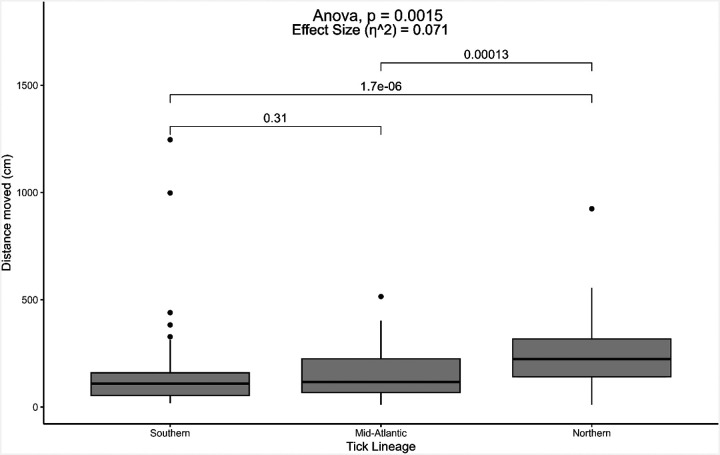
Distances moved by nymphal blacklegged ticks (*Ixodes scapularis*) from different lineages found in the United States. The “southern” lineage refers to ticks from the Oklahoma State University Tick Lab, with ticks collected from Oklahoma. The “Mid-Atlantic” lineage includes ticks collected from field locations in central Pennsylvania. The “northern” lineage refers to ticks from BEI Resources that were started from ticks collected in Rhode Island. Results from one-way ANOVA and post-hoc pairwise comparisons with Tukey’s HSD are provided. N = 58 ticks (southern), 60 (Mid-Atlantic), 60 (northern).

Overall, the distance traveled by each nymphal *I. scapularis* lineage was significantly different (p < 0.01, df = 2) (Fig. 1). In post-hoc analyses, ticks associated with the northern lineage traveled significantly farther in one hour (mean ± SE = 248.4 cm ± 20.8 cm) compared to ticks from the southern or Mid-Atlantic lineages (p < 0.01). Ticks with southern or Mid-Atlantic lineages traveled similar distances (p = 0.31), traveling approximately 155.5 ± 27.2 cm and 150.7 ± 14.4 cm, respectively, during the observation period.

**Figure 2:**
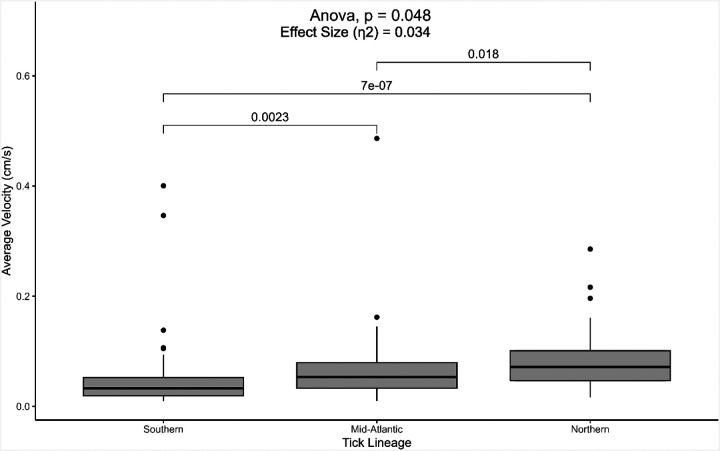
Average velocities of nymphal blacklegged ticks (*Ixodes scapularis*) from different lineages found in the United States. The “southern” lineage refers to ticks from the Oklahoma State University Tick Lab, with ticks collected from Oklahoma. The “Mid-Atlantic” lineage includes ticks collected from field locations in central Pennsylvania. The “northern” lineage refers to ticks from BEI Resources that were started from ticks collected in Rhode Island. Results from one-way ANOVA and post-hoc pairwise comparisons with Tukey’s HSD are provided. N = 58 ticks (southern), 60 (Mid-Atlantic), 60 (northern).

Like distances traveled, the average velocities of nymphal *I. scapularis* from various populations were significantly different (p = 0.05, df = 2) (Fig. 2). Nymphs from the northern lineage traveled at a higher velocity (0.078 ± 0.006 cm/s) compared to ticks with southern (p < 0.01, 0.050 ± 0.009 cm/s) and Mid-Atlantic lineages (p = 0.02, 0.064 ± 0.008 cm/s). Furthermore, nymphal ticks from the Mid-Atlantic had higher velocities compared to those from the south (p < 0.01).

**Figure 3:**
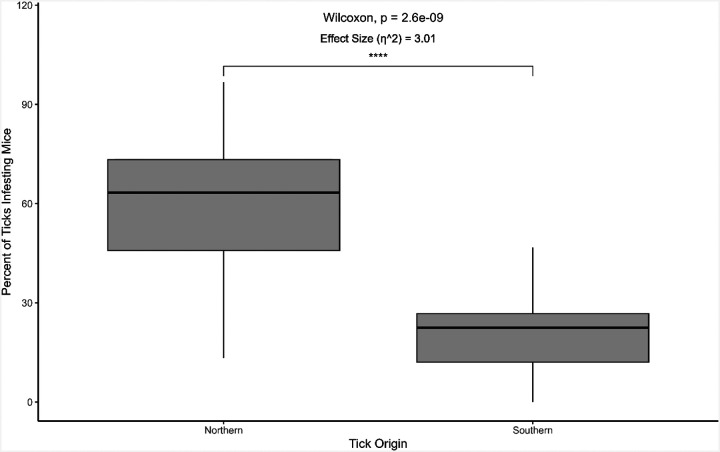
Percentage of nymphal blacklegged ticks (*Ixodes scapularis*) from different lineages found in the United States that successfully infested white-footed mice after artificial infestation in a sock. The percentage was calculated as the number of ticks that were found on the mouse after the artificial infestation period divided by the number of ticks that were added to the sock with the mouse. The “northern” lineage refers to ticks from BEI Resources that were started from ticks collected in Rhode Island. The “southern” lineage refers to ticks from the Oklahoma State University Tick Lab, with ticks collected from Oklahoma. Results from the Wilcoxon signed rank test are provided. N = 44 mice (21 male, 23 female) (northern), 22 mice (12 male, 10 female) (southern).

**Figure 4:**
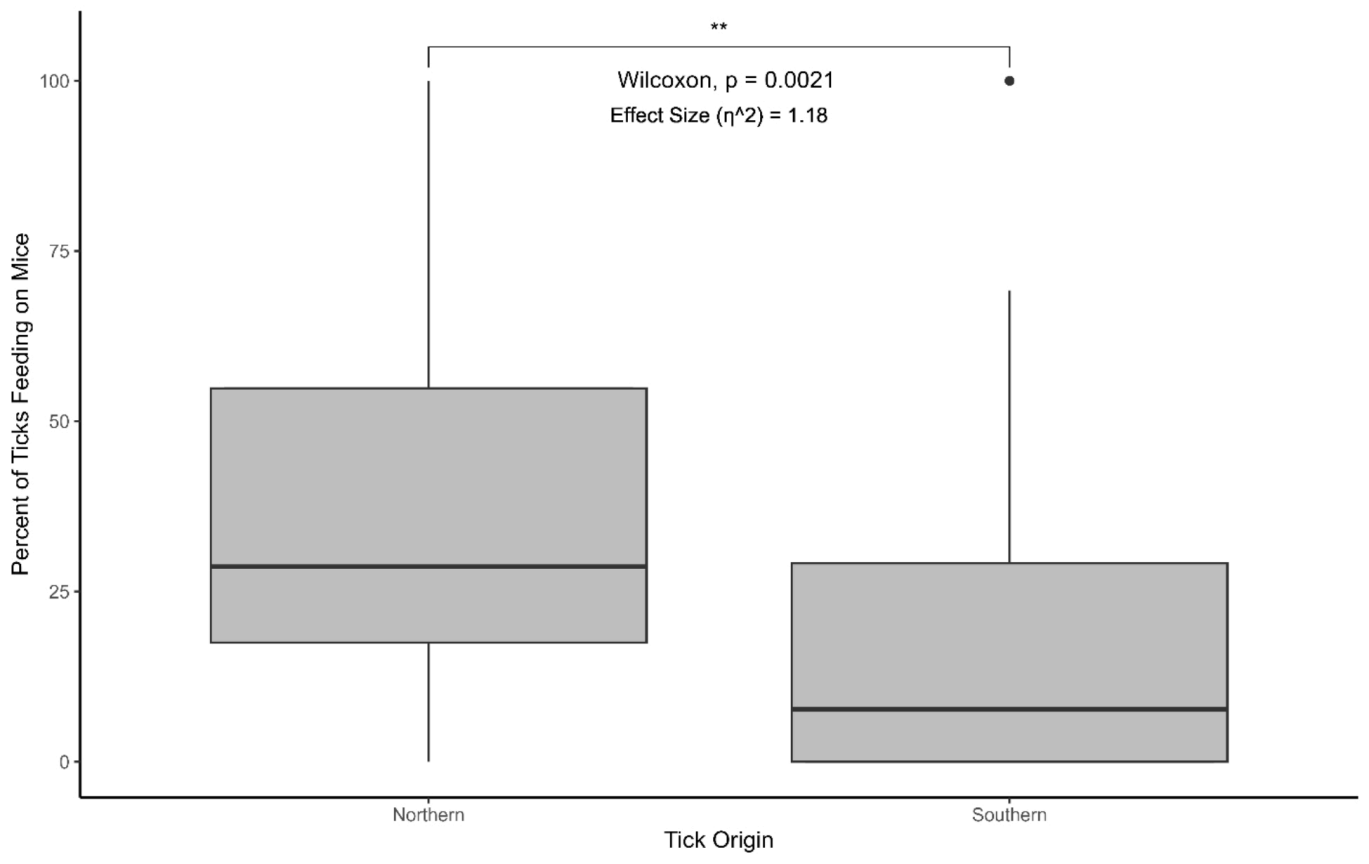
Percentage of nymphal blacklegged ticks (*Ixodes scapularis*) from different lineages found in the United States that successfully fed to repletion on white-footed mice after artificial infestation in a sock. The percentage was calculated as the number of ticks that fed to repletion on the mouse divided by the number of ticks that successfully infested the mouse after artificial infestation. The “northern” lineage refers to ticks from BEI Resources that were started from ticks collected in Rhode Island. The “southern” lineage refers to ticks from the Oklahoma State University Tick Lab, with ticks collected from Oklahoma. Results from the Wilcoxon signed rank test are provided. N = 44 mice (21 male, 23 female) (northern), 22 mice (12 male, 10 female) (southern).

While the movement experiments were done in the absence of host signals and odors, which could influence movement patterns, the infestation and feeding trials illuminated significant differences between the northern and southern lineages of *I. scapularis* in the presence of a host. The northern population of *I. scapularis* were more likely to successfully infest white-footed mice during the artificial infestation process compared to ticks from the southern population, with 60.4% ± 18.7% and 22.3% ± 13.0% of ticks successfully infesting mice, respectively (p < 0.01, W = 921.5) (Fig. 3). Of the ticks that successfully infested mice, northern lineage ticks were more likely to feed to repletion compared to southern lineage ticks (35.2% ± 26.4% and 18.5% ± 27.1 of ticks that successfully infested mice feeding to repletion, respectively) (p < 0.01, W = 709.5) (Fig. 4).

## Discussion

It is important to understand the behavior of the target animal when designing behavioral experiments. In this study, we demonstrated significant differences in movement among three lineages of blacklegged ticks and feeding behavior between the northern and southern lineages.

Our data corroborate previous studies, where ticks from a northern lineage tended to move farther distances and at faster velocities than those from a southern lineage (Arsnoe et al. 2015, 2019, Tietjen et al. 2020). Interestingly, Mid-Atlantic ticks were more similar in movement behavior to the southern population of *I. scapularis* compared to those from the northern population, even though Mid-Atlantic ticks would geographically be classified as members of the northern population (east and north of the Chesapeake Bay) (Ginsberg et al. 2014). The Mid-Atlantic ticks were collected from central Pennsylvania, which borders the proposed division between northern and southern blacklegged tick populations, emphasizing the need for additional research to define these populations.

It is possible that Mid-Atlantic ticks could be a hybrid of both northern and southern lineage ticks, where the Mid-Atlantic ticks exhibit shared characteristics or genes from both lineages. This is supported by evidence that *I. dammini* in the north (before being reclassified as *I. scapularis*) and *I. scapularis* from the south readily hybridize in the laboratory (James and Oliver 1990). Genetically, Sakamoto et al. (2014) identified two distinct clades of ticks in the United States, one that includes ticks from the northern and southern collection region and another clade that only includes ticks collected from the south. While the northern and southern lineages may have distinct demographic histories, the authors’ data support a previous hypothesis that *I. scapularis* may have originated in the south and a small founder population migrated to the north during the Pleistocene era (Norris et al. 1996, Van Zee et al. 2013, Sakamoto et al. 2014). This series of events could possibly explain the behavioral differences of all three lineages, where the Mid-Atlantic ticks could be related to both the northern and southern lineages.

Another possible explanation for differences in movement between the Mid-Atlantic and the northern lineages could be due to the effects of colonization, where behavioral differences can arise if they are raised in colony (northern lineage) versus the field (Mid-Atlantic). Differences in behavior and physiology have been reported in several insects and arthropods. For example, anthrophilic mosquitoes that are laboratory-reared may change host preferences if they are habitually fed blood from a non-human source, thereby adapting to a new host (Laarman 1958, Spitzen and Takken 2005). Behavioral differences between laboratory-reared and field-collected jumping spiders have also been reported, where the authors argued that the developmental histories of animals can affect how they respond to treatments in the laboratory (Wiggins et al. 2018). Changes in tick physiology due to differences in developmental history have also been reported. Field-caught *Dermacentor variabilis* were found to be more tolerant of dehydration compared to laboratory-reared ticks since field-caught ticks are more likely to undergo selective pressure due to less than optimal rearing conditions (Yoder et al. 2012). To the authors’ knowledge, this is the first report on behavioral differences between colony-reared and field-caught *I. scapularis*. Due to significant differences between the lineages of *I. scapularis*, future behavioral and physiological research should include testing both colony-reared and field-caught ticks to validate results.

Differences in infestation and feeding success by nymphal ticks could indicate host preference in response to host availability in the respective geographic regions, where immature northern ticks parasitize small mammals such as white-footed mice, voles, or chipmunks (Piesman and Spielman 1979, Carey et al. 1980, Mather et al. 1989, LoGiudice et al. 2003), while the southern counterparts may parasitize small mammals and other animals including birds and lizards (Spielman et al. 1984, Apperson et al. 1993, Durden et al. 2002, Piesman 2002, Goddard and Piesman 2006). Preferences for certain hosts may additionally account for the differences seen in successful infestations of white-footed mice. During animal infestations in this experiment, ticks from the southern lineage rarely attached to white-footed mice, even though they are considered permissive hosts for *I. scapularis* (Anderson and Magnarelli 1980, Ostfeld et al. 1996, Brown et al. 2023, Caron-Lévesque and Careau 2023), with only 22.3% of the ticks infesting the mouse and only 18.5% of those ticks feeding successfully to repletion (Fig. 3). The results in this study deviate from results in a previous study establishing that nymphal *I. scapularis* from Great Island, Massachusetts and Statesboro, Georgia, exhibited similar feeding success rates and host preferences for both mice and lizards (James and Oliver 1990). Even when in close proximity to a host, southern *I. scapularis* rarely attached, which could be driven by preferences for certain host cues to identify their hosts, such as host odors or heat. Southern *I. scapularis* did not infest mice at the rate of the northern lineage. Still, there were some successful infestations, indicating that southern *I. scapularis* recognized that a host was available. Still, they may have shifted or adapted their host preferences to other hosts that are more abundant in their region (i.e., lizards) (Spielman et al. 1984, Tietjen et al. 2020).

Differences in heat production by hosts could additionally explain why *I. scapularis* from the southern lineage did not frequently attach to the white-footed mice, where endotherms (i.e., small mammals) may not elicit a response from ticks compared to ectotherms (i.e., lizards). One study reported that when southern *I. scapularis* were presented with a choice of a heating source or no heating source, the ticks did not show a preference for the heating element compared to the control (Otálora-Luna et al. 2022). While *I. scapularis* is a well-known vector of several pathogens in the United States, the behavioral response of *I. scapularis* to hosts is understudied, and comparing host preferences of two distinct tick lineages is even more so. Overall, tick behavior in response to host cues is complex and requires further research to understand the nuances in the ecological relationships between ticks and their hosts.

Unknown pathogen infection status is one limitation of this study. Laboratory-reared ticks have been confirmed to be pathogen-free (Salazar 2015), however, field-collected ticks from the Mid-Atlantic region were not tested for pathogens. Pathogen infection in *I. scapularis* has been found to modify some behaviors (Benelli 2020). For example, nymphal *I. scapularis* infected with *B. burgdorferi* exhibited increased phototaxis and attraction to vertical surfaces in laboratory settings, but their questing height and ability to overcome physical obstacles were not significantly different from uninfected ticks (Lefcort and Durden 1996). When infected with *Ehrlichia muris eauclairensis*, nymphal *I. scapularis* moved faster during host-seeking compared to uninfected ticks (Aspinwall et al. 2025). In a different tick-borne disease system, *Rickettsia*-infected *Dermacentor reticulatus* females exhibited higher locomotor activity, resulting in longer trajectories compared to uninfected ticks (Pipová et al. 2023). The results from the present study showed that Mid-Atlantic ticks traveled faster than the southern lineage of ticks (Fig. 2), but both lineages traveled similar distances overall (Fig. 1). In contrast, the Mid-Atlantic ticks moved more slowly and traveled shorter distances compared to northern ticks. While the literature suggests that infected *I. scapularis* move faster, infection status would not entirely explain why the Mid-Atlantic ticks were still more similar to the southern lineage (e.g., slower and traveled less distance) compared to the northern lineage of pathogen-free ticks. In addition, 20–30% of nymphal blacklegged ticks in this region are infected with tick-borne pathogens (Brown et al. 2015, Livengood et al. 2020, Schwartz et al. 2022), but a bimodal response, suggesting infected and non-infected ticks behave differently, was not demonstrated in this study. However, discrepancies in tick behavior due to infection with pathogens in tick-borne disease systems highlight the need for future work on this topic.

Another possible limitation of this study is that it is unknown whether the recorded movement is related to the effects of energy reserves on walking activity. For *I. ricinus*, ticks with higher fat content are expected to have more energy storage and thus, travel further than ticks with lower fat (Crooks and Randolph 2006). While colony-reared ticks may have more consistent ages and therefore energy storage, similar information was not available for field-collected Mid-Atlantic ticks, where differences in their life history (e.g., age, previous blood meals, distance traveled, time since last blood meal) could result in differences in energy budgets and behaviors (Alasmari and Wall 2020). While it would have been ideal to measure energy storage before the start of the trials, the current available protocols would result in specimen destruction.

This work provides additional behavioral data that support the hypothesis of at least two distinct populations of blacklegged ticks in the United States. These differences have been previously supported by genetic, behavioral, and ecological studies comparing northern and southern populations of *I. scapularis* (Norris et al. 1996, Van Zee et al. 2013, Ginsberg et al. 2014, Sakamoto et al. 2014, Arsnoe et al. 2015, 2019, Tietjen et al. 2020). Collectively, this is the first report quantifying and comparing the horizontal movement of nymphal *I. scapularis* from two laboratory-reared populations representing different geographic regions and one field population from an area bordering the two regions. Furthermore, this research provides additional information on the differences in host infestation and feeding between two laboratory-reared colonies originating from different regions of the United States. These data provide empirical results on blacklegged tick movement and feeding behaviors that can be incorporated into tick bite risk or distribution models that account for ticks from different geographic regions. These results can also provide guidance for researchers conducting tick behavioral studies, where the choice of tick sources could affect interpretation of results and conclusions.

## Data Availability

The datasets generated during and/or analyzed during the current study are available from the corresponding author on reasonable request.

## References

[1] Alasmari S, Wall R. 2020. Determining the total energy budget of the tick *Ixodes ricinus*. Experimental and Applied Acarology. 10.1007/s10493-020-00479-1 32170536

[2] Anderson JF, Magnarelli LA. 1980. Vertebrate host relationships and distribution of ixodid ticks (Acari: Ixodidae) in Connecticut, USA. Journal of Medical Entomology. 17(4):314–323. 7420362 10.1093/jmedent/17.4.314

[3] Apperson CS, Levine JF, Evans TL, 1993. Relative utilization of reptiles and rodents as hosts by immature *Ixodes scapularis* (Acari: Ixodidae) in the coastal plain of North Carolina, USA. Exp Appl Acarol. 17(10):719–731. 10.1007/BF00051830 7628223

[4] Arsnoe I, Tsao JI, Hickling GJ. 2019. Nymphal *Ixodes scapularis* questing behavior explains geographic variation in Lyme borreliosis risk in the eastern United States. Ticks and Tick-borne Diseases. 10(3):553–563. 10.1016/j.ttbdis.2019.01.001 30709659

[5] Arsnoe IM, Hickling GJ, Ginsberg HS, 2015. Different populations of blacklegged tick nymphs exhibit differences in questing behavior that have implications for human Lyme disease risk. PLoS ONE. 10(5):1–21. 10.1371/journal.pone.0127450 PMC4440738 25996603

[6] Aspinwall J, Weck B, Martins LA, 2025. Behavioral Manipulation of *Ixodes scapularis* by *Ehrlichia muris eauclairensis*: Implications for Tick-Borne Disease Transmission. 10.1101/2025.03.04.641579 PMC12239580 40422662

[7] Benelli G. 2020. Pathogens Manipulating Tick Behavior—Through a Glass, Darkly. Pathogens. 9(8):664. 10.3390/pathogens9080664 32824571 PMC7459789

[8] Brown JE, Tiffin HS, Pagac A, 2023. Differential burdens of blacklegged ticks (Ixodes scapularis) on sympatric rodent hosts. Journal of Vector Ecology. 49(1). 10.52707/1081-1710-49.1.44 38147300

[9] Brown SM, Lehman PM, Kern RA, 2015. Detection of *Borrelia burgdorferi* and *Anaplasma phagocytophilum* in the black-legged tick, *Ixodes scapularis*, within southwestern Pennsylvania. Journal of Vector Ecology. 40(1):180–183. 10.1111/jvec.12148 26047199

[10] Carey AB, Krinsky WL, Main AJ. 1980. *Ixodes dammini* (Acari: Ixodidae) and associated ixodid ticks in South-central Connecticut, USA. Journal of Medical Entomology. 17(1):89–99. 10.1093/jmedent/17.1.89 7365754

[11] Caron-Lévesque M, Careau V. 2023. Of mice, ticks, and fleas: host behaviour and co-occurring parasites. Can. J. Zool. 101(7):510–521. 10.1139/cjz-2022-0107

[12] Crooks E, Randolph SE. 2006. Walking by *Ixodes ricinus* ticks: Intrinsic and extrinsic factors determine the attraction of moisture or host odour. Journal of Experimental Biology. 209(11):2138–2142. 10.1242/jeb.02238 16709915

[13] Curtis TR, Shi M, Qiao X. 2020. Patience is not always a virtue: effects of terrain complexity on the host-seeking behaviour of adult blacklegged ticks, *Ixodes scapularis*, in the presence of a stationary host. Medical and Veterinary Entomology. 34:309–315. doi: 10.1111/mve.12440 32227497

[14] Durden LA, Jr JHO, Banks CW, 2002. Parasitism of lizards by immature stages of the blacklegged tick, *Ixodes scapularis* (Acari, Ixodidae). Experimental and Applied Acarology. 26:257–266. 10.1023/A:1021199914816 12537298

[15] Eisen RJ, Eisen L. 2018. The Blacklegged Tick, *Ixodes scapularis*: An Increasing Public Health Concern. Trends in Parasitology. 34(4):295–309. 10.1016/j.pt.2017.12.006 29336985 PMC5879012

[16] Ginsberg HS, Rulison EL, Azevedo A, 2014. Comparison of survival patterns of northern and southern genotypes of the North American tick *Ixodes scapularis* (Acari: Ixodidae) under northern and southern conditions. Parasit Vectors. 7(1):394. 10.1186/1756-3305-7-394 25160464 PMC4153913

[17] Goddard J. 1993. Ecological Studies of *Ixodes scapularis* (Acari: Ixodidae) in Central Mississippi: Lateral Movement of Adult Ticks. Journal of Medical Entomology. 30(4):824–826. 10.1093/jmedent/30.4.824 8360912

[18] Goddard J, Piesman J. 2006. New records of immature Ixodes scapularis from Mississippi. Journal of Vector Ecology. 31(2):421–422. 10.3376/1081-1710(2006)31[421:NROIIS]2.0.CO;2 17249363

[19] Hassett E, Diuk-Wasser M, Harrington L, 2022. Integrating tick density and park visitor behaviors to assess the risk of tick exposure in urban parks on Staten Island, New York. BMC Public Health. 22:1–16. 10.1186/s12889-022-13989-x 35999523 PMC9396585

[20] James AM, Oliver JH. 1990. Feeding and Host Preference of Immature *Ixodes dammini*, *I. scapularis*, and *I. pacificus* (Acari: Ixodidae). Journal of Medical Entomology. 27(3). 10.1093/jmedent/27.3.324 2332877

[21] Laarman JJ. 1958. The host-seeking behaviour of anopheline mosquitoes. Tropical and Geographical Medicine. 10(4):293–305. 13635845

[22] Lefcort H, Durden LA. 1996. The effect of infection with Lyme disease spirochetes (*Borrelia burgdorferi*) on the phototaxis, activity, and questing height of the tick vector *Ixodes scapularis*. Parasitology. 113(2):97–103. 10.1017/S0031182000066336 8760310

[23] Levin ML, Schumacher LBM. 2016. Manual for maintenance of multi-host ixodid ticks in the laboratory. Exp Appl Acarol. 70(3):343–367. 10.1007/s10493-016-0084-8 27651325

[24] Livengood J, Hutchinson ML, Thirumalapura N, 2020. Detection of *Babesia, Borrelia, Anaplasma, and Rickettsia* spp. in Adult Black-Legged Ticks (*Ixodes scapularis*) from Pennsylvania, United States, with a Luminex Multiplex Bead Assay. Vector-Borne and Zoonotic Diseases. 10.1089/vbz.2019.2551 31976829

[25] LoGiudice K, Ostfeld RS, Schmidt KA, 2003. The ecology of infectious disease: Effects of host diversity and community composition on Lyme disease risk. Proceedings of the National Academy of Science. 100(2):567–571. 10.5586/aa.2009.011 PMC141036 12525705

[26] Marshall DS, Poh KC, Reichard MV, 2025. Spatial and temporal activity patterns of *Amblyomma americanum*. Parasites and Vectors. 18(12):1–10. 39819362 10.1186/s13071-025-06661-x PMC11740481

[27] Mather TN, Wilson ML, Moore SI, 1989. Comparing the relative potential of rodents as reservoirs of the Lyme disease spirochete (*Borrelia burgdorferi*). American Journal of Epidemiology. 130(1):143–150. 2787105 10.1093/oxfordjournals.aje.a115306

[28] Norris DE, Klompen JSH, Keirans JE, 1996. Population Genetics of *Ixodes scapularis* (Acari: Ixodidae) Based on Mitochondrial 16S and 12S Genes. Journal of Medical Entomology. 33(1):78–89. 10.1093/jmedent/33.1.78 8906909

[29] Ostfeld RS, Miller MC, Hazler KR. 1996. Causes and Consequences of Tick (*Ixodes scapularis*) Burdens on White-Footed Mice (*Peromyscus leucopus*). Journal of Mammalogy. 77(1):266–273. 10.2307/1382727

[30] Otálora-Luna F, Dickens JC, Brinkerhoff J, 2022. Behavior of Nymphs and Adults of the Black-Legged Tick *Ixodes scapularis* and the Lone Star Tick *Ambylomma americanum* in Response to Thermal Stimuli. Insects. 13(2):130. 10.3390/insects13020130 35206704 PMC8876853

[31] Piesman J. 2002. Ecology of *Borrelia burgdorferi* sensu lato in North America. In: Gray J, Kahl O, Lane RS, , editors. Lyme Borreliosis: Biology, Epidemiology, and Control. 1st ed. Wallingford, United Kingdom: CABI Publishing. p. 223–249. [accessed 2025 Mar 12]. https://www.cabidigitallibrary.org/doi/abs/10.1079/9780851996325.0000.

[32] Piesman J, Spielman A. 1979. Host-Associations and Seasonal Abundance of Immature *Ixodes dammini* in Southeastern Massachusetts. Annals of the Entomological Society of America. 72:829–832. 10.1093/aesa/72.6.829

[33] Pipová N, Peňazziová K, Baňas M, 2023. The Behavior of *Rickettsia*-Positive *Dermacentor reticulatus* Ticks under Laboratory Conditions. Life. 13(3):612. 10.3390/life13030612 36983768 PMC10056523

[34] Rosenberg R, Lindsey NP, Fischer M, 2018. Vital Signs: trends in reported vectorborne disease cases — United States and territories, 2004–2016. Morbidity and Mortality Weekly Report. 67(17):496–501. 10.15585/mmwr.mm6717e1 29723166 PMC5933869

[35] Sakamoto JM, Goddard J, Rasgon JL. 2014. Population and Demographic Structure of *Ixodes scapularis* Say in the Eastern United States. Brissette CA, editor. PLoS ONE. 9(7):e101389. 10.1371/journal.pone.0101389 25025532 PMC4099084

[36] Salazar JL. 2015. Detection of tick-borne pathogens in lab reared tick colonies and wild populations [M.S.]. Oklahoma State University. [accessed 2025 Mar 14]. https://www.proquest.com/docview/1820918743/abstract/65C4608172314808PQ/1.

[37] Schulze TL, Jordan RA. 2003. Meteorologically Mediated Diurnal Questing of *Ixodes scapularis* and *Amblyomma americanum* (Acari: Ixodidae) Nymphs. Journal of Medical Entomology. 40(4):395–402. 10.1603/0022-2585-40.4.395 14680102

[38] Schulze TL, Jordan RA, Hung RW. 2001. Effects of Selected Meteorological Factors on Diurnal Questing of *Ixodes scapularis* and *Amblyomma americanum* (Acari: Ixodidae). J Med Entomol. 38(2):318–324. 10.1603/0022-2585-38.2.318 11296842

[39] Schwartz S, Calvente E, Rollinson E, 2022. Tick-Borne Pathogens in Questing Blacklegged Ticks (Acari: Ixodidae) From Pike County, Pennsylvania. Rich S, editor. Journal of Medical Entomology. 59(5):1793–1804. 10.1093/jme/tjac107 35920050 PMC9473652

[40] [Software] R Core Team. 2024. R: A language and environment for statistical computing. http://www.r-project.org/.

[41] Spielman A, Levine JF, Wilson ML. 1984. Vectorial Capacity of North American *Ixodes* Ticks. The Yale Journal of Biology and Medicine. 57:507–513. 6516453 PMC2590044

[42] Spitzen J, Takken W. 2005. Malaria mosquito rearing – maintaining quality and quantity of laboratory-reared insects. Proceedings of the Netherlands Entomological Society Meeting. 16:95–100.

[43] Tietjen M, Esteve-Gasent MD, Li AY, 2020. A comparative evaluation of northern and southern *Ixodes scapularis* questing height and hiding behavior in the United States. Parasitology.:1–34. 10.1017/s003118202000147x PMC7677222 32772958

[44] Troughton DR, Levin ML. 2007. Life Cycles of Seven Ixodid Tick Species (Acari: Ixodidae) Under Standardized Laboratory Conditions. Journal of Medical Entomology. 44(5):732–740. 17915502 10.1603/0022-2585(2007)44[732:lcosit]2.0.co;2

[45] Van Zee J, Black WC, Levin M, 2013. High SNP density in the blacklegged tick, *Ixodes scapularis*, the principal vector of Lyme disease spirochetes. Ticks and Tick-borne Diseases. 4(1–2):63–71. 10.1016/j.ttbdis.2012.07.005 23219364

[46] Wiggins WD, Bounds S, Wilder SM. 2018. Laboratory-reared and field-collected predators respond differently to same experimental treatments. Behav Ecol Sociobiol. 72(2). 10.1007/s00265-017-2437-7

[47] Yoder JA, Hedges BZ, Benoit JB. 2012. Water balance of the American dog tick, *Dermacentor variabilis*, throughout its development with comparative observations between field-collected and laboratory-reared ticks. International Journal of Acarology. 38(4):334–343. 10.1080/01647954.2011.647073

